# Behavioral signs of CHARGE syndrome and CHD7 mutational spectrum

**DOI:** 10.1192/j.eurpsy.2023.767

**Published:** 2023-07-19

**Authors:** N. Bouayed Abdelmoula, B. Abdelmoula

**Affiliations:** Genomics of Signalopathies at the service of Medicine, Medical University of Sfax, Sfax, Tunisia

## Abstract

**Introduction:**

CHARGE syndrome is a genetic entity caused by mutations in the chromodomain helicase DNA-binding protein 7 gene (CHD7) at 8q12.1. There are pleiotropic signs among individuals with this disorder. Diagnosis is clinical using medical criteria. CHD7 gene mutations are usually found in 90% of affected patients.

**Objectives:**

The aim of this study was to report behavioral signs of CHARGE syndrome and their phenotype-genotype correlations.

**Methods:**

Four Tunisian males from Sfax (Tunisia) with clinical features suggestive of CHARGE syndrome were examined at our genetic counselling at the medical University of Sfax. Assessment of facial dysmorphic and behavioral features, karyotyping using RHG banding and molecular screening of CHD7 mutations were performed. Molecular analysis was made using direct Sanger sequencing of the entire CHD7 gene.

**Results:**

Molecular genetic analysis revealed two deletions of the CHD7 gene at exon 3 for the first patient and at exon 8 for the second. The two genetic alterations were associated to retarded growth development and genital hypoplasia. Sensory impairments included for the first visual defects and for the second auditory and olfactory defects. Besides constant delayed psychomotor development, the two patients shared receptive and expressive communication disorders, anxiety, attention deficit, cognitive impairment and intellectual disability. There were no aggressive traits nor major autistic features. Learning disabilities were also present for the two patients.

**Conclusions:**

The CHD7 gene controls the developmental pathways as a transcriptional regulator in the nucleoplasm through chromatin organization. Mutational alterations lead according to the affected domains, and the structure of the nonfunctional CHD7 protein, to the perturbation of the regulation of the developmental pathways’ genes expression. CHD7 is demonstrated as an important component of neurogenesis through two neuronal determination factors: Sox4 and Sox11. While nonsense, frameshift and missense mutations are most common, deletions and duplications are less frequent. Moreover, while exon 3 is commonly altered, mutations of exon 8, which is related to the CHD7 protein chromodomain, are very rare. Phenotype-genotype correlations according to the type of genomic alteration of CHD7 gene are rarely published, particularly concerning behavioral and psychological features of CHARGE association. Here, physical disorders of our two patients seem to be different but behavioral features seem to be common. Multidisciplinary care is thus required for CHARGE syndrome and molecular analysis must be indicated because the type of the genomic alterations may be a key step for a more accurate management of physical and behavioral disorders.

**Disclosure of Interest:**

None Declared

